# Accuracy and effectiveness of thoracolumbar pedicle screw placement with a mixed reality head mounted device: first clinical experience and results

**DOI:** 10.1016/j.bas.2026.106022

**Published:** 2026-03-26

**Authors:** Bernardo Reyes Medina, Fatemeh Khafaji, Safwan Saffour, Christoph Sippl, Stefan Linsler

**Affiliations:** Klinik für Neurochirugie, Klinikum Bayreuth, Institut für Lehre und Forschung Medizincampus Oberfranken MCO, Friedrich-Alexander-Universität Erlangen-Nürnberg, 95455 Bayreuth, Deutschland

**Keywords:** Techniques and innovations, Spine surgery, Mixed reality, Augmented reality, Navigation, Thoracolumbar screw placement, Head mounted device

## Abstract

**Introduction:**

Mixed reality (MR) has the potential to enhance the accuracy and effectiveness of spine surgery, particularly in the placement of pedicle screws. This technology can lead to improved outcomes, optimized ergonomics, and reduced operative time and costs.

**Research question:**

This study evaluates the efficacy and accuracy of MR in thoracolumbar pedicle screw placement using neuronavigation and a head-mounted device.

**Material and methods:**

We conducted a prospective review of the initial series of thoracolumbar pedicle screw placements utilizing neuronavigation (Brainlab) and MR with a head-mounted device (MagicLeap2). Data from 22 patients were analyzed, involving a total of 144 pedicle screws placed. The Gertzbein-Robbins classification (GRC) was used to assess screw position: Grade A (screw within the pedicle), Grade B (breach <2 mm), Grade C (breach 2-4 mm), Grade D (breach 4-6 mm), and Grade E (breach >6 mm). Grades A and B were considered accurate placements.

**Results:**

Out of 144 thoracolumbar pedicle screws, 139 were accurately positioned according to GRC grades A and B, resulting in a clinical accuracy rate of 96.5%. The mean deviation at the bone entry point compared to preoperative plans was 2.35 ± 1.1 mm, with a mean angular deviation of 3.1 ± 1.4°. Surgeons reported no issues with the head-mounted device, including dizziness or headaches, and noted no limitations in visualization of the surgical field.

**Discussion and conclusion:**

This study demonstrates the successful application of MR combined with neuronavigation for precise pedicle screw placement, eliminating the need for fluoroscopy and enhancing surgical efficiency.

## Highlights

• First clinical evaluation of an AR head-mounted device combined with a conventional navigation system.
• Clinical accuracy rate was 96.5% for pedicle screw placement, with 139 out of 144 screws.
• AR-HMD provides visual guidance for identifying entry points, determining skin incisions.
• Use of the HMD allowed surgeons to maintain focus on the surgical field.

## Abbreviation list:

AR: augmented reality
CT: computer tomography
VR: virtual reality
CBCT: intraoperative 3 D Scan
GRC: Gertzbein-Robbins classification
HMD: head mounted device
MISS: minimally invasive spine surgery

## 1 Introduction/background

Over the last years modern technologies in spine surgery have been developed. The use of augmented reality (AR) and mixed reality (MR) have become useful in the neurosurgery, especially in medical education, surgical planning and intraoperative navigation (Mishra et al., 2022; Morimoto et al., 2022; Yeung et al., 2021). AR superimposes computer generated virtual objects onto the users’ view of the real world (Guha et al., 2017; Azuma, 1997), whereas MR is an extension of AR, which allows interaction between virtual objects and real world elements, allowing feedback interaction (Cho et al., 2020; Zhang et al., 2021; Zari et al., 2023; Wilson et al., 2025).

To assess the accuracy of pedicle screw placement, the Gertzbein-Robbins classification (GRC) was developed (Gertzbein and Robbins, 1990). The accuracy of the free hand fluoroscopy placement of pedicle screws in the thoracolumbar spine has been reported from 65% to 93% (Li et al., 2023a; Yan et al., 2022; Marcus et al., 2014), whereas the placement of pedicle screws with the use of image based-neuronavigation has reported better rates of accuracy up to 95% (Shree Kumar et al., 2020; Molliqaj et al., 2017; Staartjes et al., 2018) and with robotics systems similar or even better.

MR benefits directly with the implementation of head mounted devices (HMDs), allowing the user project a hologram and project into the patient during spine surgery (Aoyama et al., 2022a, 2022b; Jud et al., 2020). This use of HMD-MR based navigation is an extremely useful tool in spine surgery (Dibble and Molina, 2021). HMDs maintain an unaltered direct view of the real world and parallelly project a virtual 2D or 3D model of the region of interest (Zari et al., 2023).

Some cadaveric and model studies have evaluated the accuracy of using head-mounted displays (HMD) with mixed reality (MR) in the placement of pedicle screws in both the thoracolumbar and cervical spine (Cao et al., 2023; Ruiz-Cardozo et al., 2024; Frisk et al., 2022; Móga et al., 2023; Li et al., 2023b; Winkler et al., 2024; Ghenbot et al., 2024). Augmented reality provides a significant advantage, enabling even inexperienced surgeons to train effectively, thereby establishing an important tool for surgical education (Ghenbot et al., 2024). However, the benefits of HMD-MR-based navigation in terms of accuracy for pedicle screw implantation in the thoracolumbar or cervical spine, as well as its impact on surgical time and radiation exposure, have not yet been thoroughly evaluated (Móga et al., 2023; Burström et al., 2021). First results in cadaver and models showed promising results using AR- and MR-head mounted devices for placement of pedicle screw with high accuracy with over 95% ideal placement (Frisk et al., 2022). To date, there are very few reports concerning the use of HMD with MR in spine surgery and the subsequent clinical outcomes for patients (Felix et al., 2022). This article presents the authors' initial results from a new HMD system and discusses the surgical outcomes observed in a single-center experience.

## 2 Material and methods

### 2.1 Patient criteria

The authors prospectively analyzed data from their first twenty-two patients with thoracolumbar pathologies, including traumatic and degenerative conditions, who underwent pedicle screw fixation of the thoracolumbar spine (see Table 1).

**Table 1:**
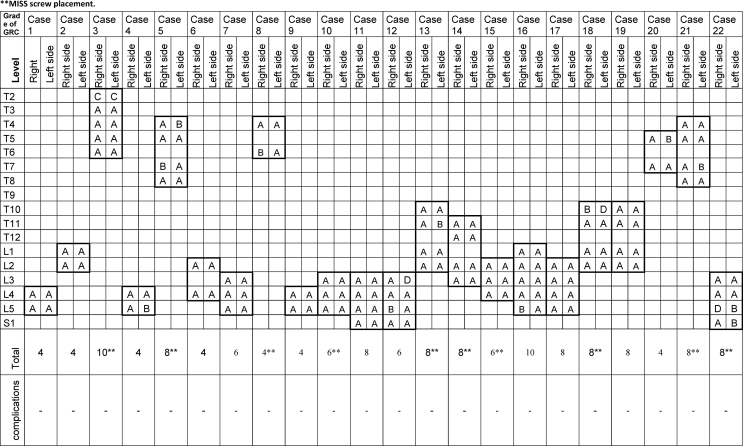
Patients, vertebral level of the implanted pedicle screws and their respective GRC grade.

The inclusion criteria were as follows: patients over 18 years of age who underwent posterior thoracic and/or lumbar fusion between T1 and S1. Additionally, CT images capable of 3D modeling were required for preoperative planning of screw placement. The exclusion criteria included: patients under 18 years, pregnant patients.

The study was conducted at the Department of Neurosurgery, Bayreuth Hospital, Medical Campus of Friedrich-Alexander-University Erlangen-Nuremberg, Upper Franconia. Ethical approval was obtained from the Ethics Committee of Friedrich-Alexander-University Erlangen-Nuremberg (No. 25-20 Bm). The study was conducted in accordance with the Declaration of Helsinki. Written informed consent was obtained from all patients.

Surgical procedures were performed either using an open approach or a minimally invasive spine surgery (MISS) technique, depending on the underlying pathology and preoperative planning decisions. Comprehensive data were collected, including demographic characteristics, underlying pathology, intraoperative findings, registration time, screw placement time, and procedure-related complications. In addition, surgeon-reported outcomes were assessed, including comfort and perceived constraints associated with the head-mounted device (HMD), occurrence of discomfort (e.g., headache or dizziness), and any limitations in visualization of the surgical field.

### 2.2 Intraoperative registration and surgical technique with navigation and use of mixed reality

**Fig. 1:**
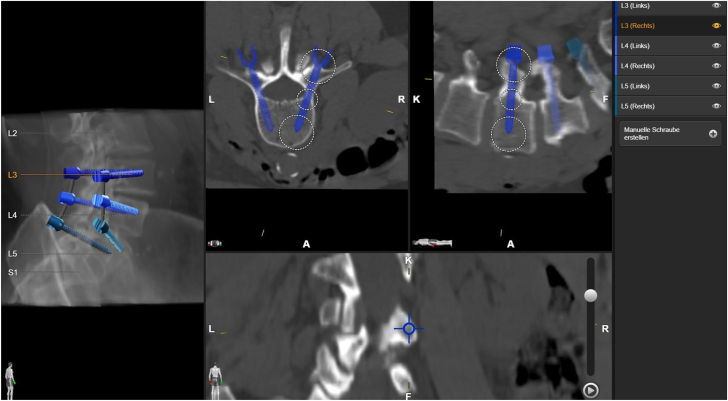
Exemplary case of preoperative planning of screw placement in navigation software.

Preoperatively, CT imaging of the spine was performed to plan thoracolumbar pedicle screw trajectories (Fig. 1) using a dedicated navigation planning system (Elements Spine Planning, Brainlab, Germany). On the day prior to surgery, each pedicle screw trajectory was individually assessed and refined by the neurosurgeon within the navigation software (Elements Spine Planning, Brainlab, Germany). The finalized planning data were stored for intraoperative use. Planning required approximately 4–7 min, depending on the number of screws.

Procedures were conducted in a hybrid operating room equipped with a C-arm system (Cios Spin, Siemens Healthcare, Germany) enabling intraoperative cone beam CT (CBCT) imaging. Augmented reality (AR) navigation software compatible with the Magic Leap 2 head-mounted device (HMD) (Magic Leap, FL, USA) was integrated into the Brainlab navigation platform and utilized in all cases.

Following induction of general anesthesia, patients were positioned prone on the operating table, and standard antiseptic skin preparation was performed. Fluoroscopy was used to identify the lowest vertebral level of the planned instrumentation. Surgical exposure was carried out at the predetermined vertebral levels. A radiolucent reference clamp with four reflective spheres was secured to the spinous process of the lowest instrumented vertebra to serve as a neuronavigation reference.

An intraoperative CBCT scan was acquired to enable automatic image co-registration. Preoperative CT datasets and planned screw trajectories were fused with the CBCT images to generate a high-resolution three-dimensional (3D) model of the spine. Given the prone positioning and stabilization with dense foam padding to minimize intervertebral motion, rigid image fusion was considered sufficient for accurate alignment of pre- and intraoperative datasets. Navigation accuracy was verified by correlating anatomical landmarks with the navigation reference.

**Fig. 2:**
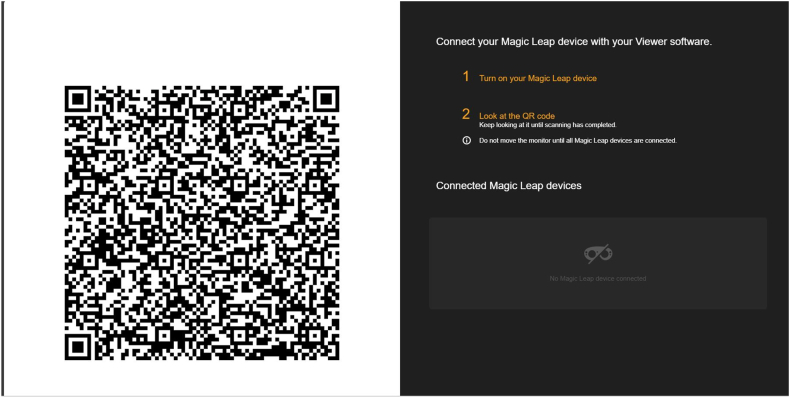
QR code generated from Brainlab navigation system to match the head mounted device (HMD) Magic leap 2 with the navigation system intraoperatively.

The Colibri drill guide (Brainlab, Munich, Germany) and screwdriver (DePuy Synthes, Raynham, MA, USA) were registered within the navigation system. The Magic Leap 2 HMD was subsequently synchronized with the Brainlab navigation platform via QR code (Fig. 2).

The Magic Leap 2 device combines augmented and mixed reality technologies, projecting two- and three-dimensional navigational information directly into the surgeon's field of view. Interaction with virtual models was enabled through gesture-based controls.

Registration time was recorded and defined as the interval between completion of CBCT acquisition and successful visualization of navigational data and the operative field within the HMD display. Support staff were needed to assist with putting on the HMD; no consumables are required.

**Fig. 3:**
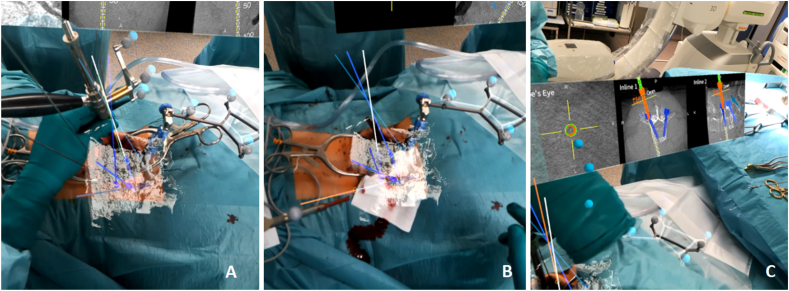
Exemplary case of lumbar spine surgery with surgeon's view through the MagicLeap2 device: A) and B) mixed reality with spine model and screw planning and their trajectories in L3, L4 and L5; C) augmented reality with visualization of CT scan with preplanned screws and real time navigated screw placement.

**Fig. 4:**
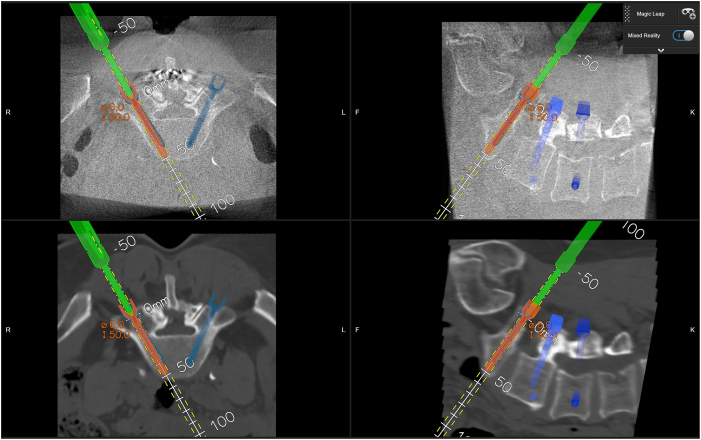
Exemplary case of lumbar spondylodesis L4 to S1 with planed screw trajectory (in blue) and real time navigated screw placement in overlay view (orange).

Following HMD registration, pedicle screws were inserted using a fluoroscopy-free four-hand technique. One surgeon aligned the drill guide according to the projected mixed reality trajectory corresponding to the preplanned screw path (Fig. 3). The second surgeon performed pedicle cannulation using the Colibri system through the drill guide. A K-wire was inserted, followed by placement of the preplanned pedicle screw (Fig. 4).

The surgical workflow and mixed reality visualization are demonstrated in the supplementary video.

After screw insertion, a confirmatory CBCT scan was performed to assess implant positioning. Malpositioned screws were revised as required. Rods were subsequently inserted according to the preoperative plan.

Clinical follow-up was conducted for a minimum of three months post-discharge.

### 2.3 Radiographic analysis of placed pedicle screws

Screw placement accuracy was assessed using postoperative computed tomography (CT) imaging according to the Gertzbein–Robbins classification (GRC): Grade A (completely intrapedicular), Grade B (cortical breach <2 mm), Grade C (breach 2–4 mm), and Grade D (breach 4–6 mm), and Grade E (breach >6 mm). Grades A and B were defined as clinically acceptable screw placement.

Quantitative accuracy analysis was performed by comparing the preoperatively planned screw trajectory with the actual screw position. Deviations were measured in millimeters (mm) at both the pedicle entry point and the screw tip. In addition, angular deviation in the axial plane was calculated (Fig. 4).

For technical accuracy assessment, three-dimensional (3D) measurements were obtained by fusing intraoperative imaging data with the preoperative planning dataset. Linear deviations at the entry point and tip, as well as angular deviations, were derived from the fused datasets.

### 2.4 Statistical analysis

The analysis of data was performed using SPSS (SPSS, version 22, IBM Corporation, NY, US). The different grades of GRC were obtained and were calculated using a simple ratio method and were obtained for the overall sample. Significance level was set at p < 0.05. Values are presented as mean ± standard deviation.

## 3 Results

### 3.1 General results

Between February and June 2025, twenty-two patients underwent thoracolumbar spine surgery using MR guidance with a head-mounted device (HMD) at the Neurosurgical Department of Klinikum Bayreuth, Medical Campus Oberfranken, performed by two experienced spine surgeons (SL, CS). The cohort included 10 male (45.5%) and 12 female (54.5%) patients, with a mean age of 67.9 ± 10 years. Spinal trauma accounted for 45.5% of cases (n = 10), while degenerative pathologies accounted for 54.5% (n = 12).

A total of 144 pedicle screws were implanted, with 4–10 screws per patient. Of these, 70 screws were placed via small skin incisions without direct exposure of the pedicle entry point, and 74 screws were placed percutaneously using minimally invasive spine surgery (MISS) techniques. Treated levels and corresponding Gertzbein–Robbins classification (GRC) grades are summarized in Table 1.

Preoperative trajectory planning required approximately 4-7 min per patient with neuronavigation software Elements Spine Planning, Brainlab, Germany. The mean time from completion of intraoperative CBCT acquisition to successful fusion with the preoperative plan and visualization on the HMD was 204 ± 22 s. Thereby, the connection of the HMD to navigation system required in mean 25 ± 12 s.

Mean screw insertion time was 92 ± 24 s per screw.

No postoperative neurological deficits, infections, hemorrhages, or wound healing complications occurred. All patients were discharged within six days postoperatively and remained free of new complaints or complications during follow-up.

Clinical accuracy, assessed via GRC, demonstrated that 139 of 144 screws were accurately placed (Grades A and B), while two screws were graded C and three screws graded D. Overall clinical accuracy was 96.5%. Intraoperative 3D scanning identified the inaccurately placed screws, which were subsequently revised. Postoperative CT confirmed all screws as Grades A or B.

Linear deviation of screws at the pedicle entry point relative to the preoperative plan averaged 2.35 ± 1.1 mm, with an angular deviation of 3.1 ± 1.4°.

Surgeons reported no adverse effects from HMD use, including dizziness, headache, or neck pain. There were no limitations in surgical field visualization or disorientation caused by the augmented/mixed reality display (see Table 2). No HMD-related complications were observed.

**Table 2:** Impression of surgeons while using HMD.

	Surgeon 1	Surgeon 2	Total
Headache	0/14	0/8	0/22
Neck pain	0/14	0/8	0/22
Dizziness	0/14	0/8	0/22
Mixed reality view helpful	13/14	7/8	20/22
Augmented reality view of CT helpful	14/14	8/8	22/22
Improved surgical orientation	12/14	7/8	19/22
Complaints and intraoperative complications due to HMD	0/14	0/8	0/22

## 4 Discussion

The use of mixed reality (MR) head-mounted displays (HMDs) for pedicle screw placement in spine surgery remains a novel approach and has not yet been incorporated into routine clinical practice. Recent studies have highlighted the potential of this technology, demonstrating improved pedicle screw placement accuracy and underscoring its promise for enhancing surgical precision and potentially transforming standard practices in spine surgery (Ma et al., 2025):Projector-based AR devices has demonstrated high clinical accuracy in cadaveric models in previous publications (Cao et al., 2023; Ruiz-Cardozo et al., 2024; Winkler et al., 2024). A trial comparing surgical navigation techniques, including AR, to traditional free-hand methods is assessing the accuracy and safety of pedicle screw placement in patients undergoing spinal procedures (Ma et al., 2025; Asada et al., 2024; Gelalis et al., 2012). Initial results from cadaveric models indicate promising outcomes, with AR and mixed reality (MR) head-mounted devices achieving over 95% accuracy in ideal screw placement (Frisk et al., 2022). However, the use of MR head-mounted displays (HMDs) for pedicle screw placement in spine surgery remains a novel approach that has yet to be integrated into routine clinical practice. The authors present their first results and experience using a head-mounted device (HMD) with augmented reality (AR) and mixed reality (MR) for pedicle screw placement in combination with spinal navigation.

According to the results, the accuracy was very high and reliable, exceeding 95%. This is comparable to published literature: robotic-guided screw placement (up to 98.6% accuracy) and free-hand technique (up to 94%) (Asada et al., 2024; Gelalis et al., 2012).

The study did not reveal any disadvantages or intraoperative limitations associated with HMD use. The HMD allows the surgeon to maintain focus on the surgical field without moving their head or diverting their gaze to a monitor or other devices. This enhances the surgeon's comfort and allows greater concentration on the procedure. Furthermore, it improves workflow and may reduce overall surgical time. In the present analysis, the mean time for pedicle screw placement was 92 ± 24 s per screw. Other studies reported 130 ± 55 s in a similar setting (23), 179 ± 65 s for robotic-assisted screw placement, and 164 ± 83 s for free-hand placement (Torii et al., 2022). Connecting the MR-HMD intraoperatively was simple and fast. Once connected, screws could be placed without delay. The authors anticipate that intraoperative use of the MR-HMD will significantly reduce pedicle screw placement time and may further improve accuracy in future applications.

Using the MR model with preplanned screw trajectories also allows skin incisions and screw placement without fluoroscopy. Traditionally, fluoroscopy is used for intraoperative guidance in minimally invasive procedures, with reported accuracy ranging from 83.9% to 98% (Marcus et al., 2014; Shree Kumar et al., 2020). However, fluoroscopy exposes the surgical team to radiation. Modern imaging techniques and computer-assisted navigation can eliminate the need for intraoperative fluoroscopy, reducing radiation exposure (Li et al., 2023a; Yu and Khan, 2014; Zhang et al., 2014). In this study, no fluoroscopy was used during pedicle screw placement. Mixed reality combined with navigation thus has the potential to remove fluoroscopy entirely, reducing risks to the surgical team. Additionally, well-planned skin incisions minimize postoperative wound healing complications by reducing skin traction, contributing to better overall outcomes. This aspect should be investigated further in future studies.

Another advantage of the MR-HMD is the ability to interact directly with the navigation system, allowing the user to manipulate the 3D model, including rotation, resizing, and scrolling through CT scans via gesture control in the surgical field.

Liebmann et al. (2019) used the HoloLens to place pedicle screws in an open lumbar spine model and reported a mean deviation of 2.77 ± 1.46 mm at the entry point, with an angular deviation of 3.38 ± 1.73° (Liebmann et al., 2019). The results of the present study demonstrated comparable data. Molina et al. (2019) employed Augmedics XVision to place thoracolumbar pedicle screws in cadaveric torsos during open surgery, achieving a clinical accuracy of 94.6% according to the Gertzbein scale (Molina et al., 2019). In a minimally invasive setup, Liu et al. reported 94% accuracy using the HoloLens (Liu et al., 2020). The authors achieved comparable clinical accuracy of 96.5% with the Magic Leap 2 HMD and Brainlab spinal navigation system. The authors found that learning to use the head-mounted display (HMD) was not an issue. No learning curve was observed.

### 4.1 Limitations

Limitations of this study include the small sample size and the monocentric setting with only two experienced surgeons. Consequently, the statistical robustness of the results is limited with a smaller sample size, and outcomes may be influenced by surgeons' high experience in this cohort. However, the authors designed this study with two experienced surgeons to minimize potential bias that could arise from involving different surgeons in the initial clinical study.

Further randomized, prospective, multicentric studies with larger cohorts are needed to evaluate this promising MR/AR HMD technology and its benefit for education as well as in clinical routine for young as well as experienced spine surgeons. Nevertheless, the authors were able to demonstrate their initial experience and surgical outcomes using AR/MR HMD in patients with various lumbar and thoracic pathologies undergoing minimally invasive or open spinal procedures.

## 5 Conclusion

This study constitutes the first clinical evaluation of an augmented reality (AR) head-mounted device (HMD) integrated with a conventional navigation system for minimally invasive spinal pedicle screw placement in a small patient cohort. The AR-HMD provides visual guidance for identifying pedicle entry points, planning skin incisions, and directing instruments intraoperatively. Accurate localization of the bone entry point is critical, and the 3D mixed reality model enhances visualization of the instrument relative to the underlying anatomy, supporting adherence to the planned implant trajectory.

Although recently introduced, HMDs combined with neuronavigation and mixed reality have demonstrated notable effectiveness, accuracy, and ergonomic benefits. AR/MR guidance in minimally invasive spine surgery (MISS) holds considerable promise, driven by advances in real-time processing and increasing clinical adoption. By addressing current challenges, MR-guided pedicle screw placement has the potential to enhance surgical precision and procedural efficiency.

Further prospective, randomized studies with larger patient cohorts are required to rigorously evaluate the impact of MR/AR devices on pedicle screw placement accuracy. Future investigations should also consider the role of this technology in residency and fellowship training programs to assess its educational value.

## Informed consent statement

None.

## Author contributions

Conceptualization: Bernardo Reyes Medina, Stefan Linsler. Methodology: Bernardo Reyes Medina, Safwan Saffour, Stefan Linsler. Validation: Fatemeh Khafaji, Stefan Linsler, Christoph Sippl. Formal analysis: Christoph Sippl, Safwan Saffour, Fatemeh Khafaji. Investigation: Bernardo Reyes Medina, Stefan Linsler. Writing: Bernardo Reyes Medina, Stefan Linsler. Original draft preparation: Bernardo Reyes Medina Stefan Linsler, Fatemeh Khafaji. Review and editing: Bernardo Reyes Medina, Stefan Linsler, Safwan Saffour, Fatemeh Khafaji, Christoph Sippl. Supervision: Stefan Linsler. Project administration: Bernardo Reyes Medina, Stefan Linsler.

## Declaration of generative AI and AI-assisted technologies in the manuscript preparation process

During the preparation of this work the authors used ChatOn to edit grammar. After using this service, the authors reviewed and edited the content as needed and take full responsibility for the content of the published article.

## Funding

This research received no external funding.

## Author declaration of competing interest

I wish to draw the attention of the Editor to the following facts which may be considered as potential conflicts of interest to this work: S Linsler received honorarium for presentations from Brainlab company. Relationships did not influence the results of the presented work. Beside this, there is nothing to declare by the authors.

I confirm that the manuscript has been read and approved by all named authors and that there are no other persons who satisfied the criteria for authorship but are not listed. I further confirm that the order of authors listed in the manuscript has been approved by all of us.

I confirm that I have given due consideration to the protection of intellectual property associated with this work and that there are no impediments to publication, including the timing of publication, with respect to intellectual property.

I understand that the Corresponding Author is the sole contact for the Editorial process (including Editorial Manager and direct communications with the office). He is responsible for communicating with the other authors about progress, submissions of revisions and final approval of proofs. I confirm that I have provided a current, correct email address which is accessible by the Corresponding Author.

## Appendix A Supplementary data

**Supplementary video:** setting and surgical procedure with HMD devices in lumbar spine surgery.
